# Hyperspectral prediction of leaf area index of winter wheat in irrigated and rainfed fields

**DOI:** 10.1371/journal.pone.0183338

**Published:** 2017-08-17

**Authors:** Guangxin Li, Chao Wang, Meichen Feng, Wude Yang, Fangzhou Li, Ruiyun Feng

**Affiliations:** 1 College of Agronomy, Shanxi Agricultural University, Taigu, China; 2 Institute of Crop Science, Shanxi Academy of Agricultural Sciences, Taiyuan, China; Institute of Genetics and Developmental Biology Chinese Academy of Sciences, CHINA

## Abstract

The growth status of winter wheat in irrigated field and rainfed field are obviously different and the field types may have an effect on the predictive accuracy of hyperspectral model. The objectives of the present study were to understand the difference of spectral sensitive wavelengths for leaf area index (LAI) in two field types and realize its hyperspectral prediction. In study, a total of 31 and 28 sample sites in irrigated fields and rainfed fields respectively were selected from Wenxi County, and the LAI and canopy spectra were also collected at the main grow stage of winter wheat. The method of successive projections algorithm (SPA) was applied by selecting the important wavelengths, and the multiple linear regression (MLR) and partial least squares regression (PLSR) were used to construct the predictive model based on the important wavelengths and full wavelengths, respectively. Moreover, the parameters of variable importance project (VIP) and B-coefficient derived from PLSR analysis were implemented to validate the evaluated wavelengths using the SPA method. The sensitive wavelengths of LAI for irrigated field and rainfed field were 404, 407, 413, 417, 450, 677, 715, 735, 816, 1127 and 404, 406, 432, 501, 540, 679, 727, 779, 1120, 1290 nm, respectively, and these wavelengths proved to be highly correlated with LAI. Compared with the model performance based on the SPA-MLR and PLSR methods, the method of SPA-MLR was proved to be better (rainfed field: R^2^ = 0.736, RMSE = 1.169, RPD = 1.6245; irrigated field: R^2^ = 0.716, RMSE = 1.059, RPD = 1.538). Moreover, the predictive model of LAI in rainfed fields had a better accuracy than the model in irrigated fields. The results from this study indicated that it was necessary to classify the field type while monitoring the winter wheat using the remote sensing technology. This study also demonstrated that the multivariate method of SPA-MLR could accurately evaluate the sensitive wavelengths and construct the predictive model of LAI.

## Introduction

For the past 40 years, spectral remote sensing technology provides an effective method for large-scale, non-destructive and rapid estimation of crop yield and monitoring of growth [1–2]. The LAI is often used as an important index for reflecting crop growth condition, regulating water and fertilizer management, monitoring growth conditions and predicting yield [3–4]. Therefore, the application of spectral remote sensing technology on monitoring the LAI of crops has been potentially observed.

Feng et al. [5] evaluated the spectral characteristics of maize LAI, and constructed the spectral quantitative model of LAI under the drought stress. Zarate-Valdez et al. [6] evaluated the accurate LAI of the orchard by integrating many vegetation indices, and reported its predictive accuracy as 0.9. Zhang et al. [7] also used the hyperspectral technique to evaluate LAI. These studies indicate that it is feasible to detect high-precision prediction of LAI by using the hyperspectral technology. The winter wheat fields including irrigated field and rainfed field in northern China are very common. The water condition is the main difference between these field types and the main factor affecting the growth of winter wheat [8]. Feng et al. [9] reported that the growth of winter wheat under water and drought conditions is significantly different, and the winter wheat growth monitoring accuracy without differentiating water and drought land types is lower than that under the condition of classifying water and drought land types. Monitoring the growth status of winter wheat in larger area by using the remote sensing is a rapid and real-time technology. It is necessary to distinguish the field type of winter wheat from irrigated fields and rainfed farmland so as to improve the spectral monitoring accuracy of winter wheat growth.

Moreover, some researches pointed that extracting the spectral information and constructing the optimal predictive model is one of the main ways to effectively improve the model performance [10]. Multivariate statistical analysis methods such as successive projections algorithm (SPA), multiple linear regression (MLR) and partial linear square regression (PLSR) have been widely used in evaluating spectral information and optimizing model performance. Araújo et al. [11] reported that the SPA can overcome the collinearity between the independent variables to select the important wavelengths. At the same time, many studies combining SPA and MLR methods have achieved the target spectral information and robust model [12–14]. Currently, the PLSR method is able to compress the spectral data to less variable factors, and also overcome the collinearity between the independent variables, and can build a model of high accuracy and robustness. Hence, the PLSR method is widely used in spectral analysis [15–16].

In this study, the winter wheat in the rainfed and irrigated fields of Wenxi county, Shanxi province was used as the object, and the multivariate statistical methods of SPA, MLR and PLSR were comprehensively adopted to achieve the following objectives: (i) spectral characteristics evaluation of LAI in different types of wheat fields; (ii) determining the prediction of LAI in different types of wheat fields; (iii) to evaluate the effect of field types on the hyperspectral monitoring of LAI; (iv) analyzing the application of multivariate statistical methods on evaluating the important wavelengths corresponding to LAI and constructing the predictive models.

## Materials and methods

### Experimental site

In this study, Wenxi County, southwestern Shanxi Province (110° 59'33 "E-111° 37'29" E, 35° 9'38 "N -35° 34'11" N) was selected as the study area. It is located in the intersection of Linfen basin and Yuncheng basin, north of Huanghuai winter wheat area. According to standards of the International Council of Scientific Unions (ICSU), the soil in this area is mainly calcareous cinnamon, and the soil is neutral and slightly alkaline with more accumulation of minerals and organic matter, thicker humus layer and higher fertility. The climate of this area is a typical warm continental with large temperature difference between day and night and four distinctive seasons. In the whole county, the irrigation farmland is slightly more than the rainfed farmland. The wheat varieties (Shunmai 1718, Linfen 8050, Yannong 19 and Linfeng 3) were commonly cultivated in this area, and the growth period is from early October to early June of next year.

Winter wheat was sown on October 1 to 10, 2012, and irrigated on November 7 round about the beginning of winter. 5–7 days after irrigation, it was cultivated and compacted to promote wheat tillering and root development; On mid-February 2013 when the winter wheat turned green it was cultivated and weeded, in early March when jointing it was irrigated again, and urea was used 15 kg per mu to promote tillering; In early May it was irrigated to boost grouting which is conducive to producing more and fuller seeds. In mid-June the winter wheat was ripe for harvest.

### Experimental design

The experiment was conducted from September, 2012 to June, 2013 in the townships of Wenxi county. The rainfed and irrigated fields with uniform wheat growth were selected according to their distribution characteristics in the region. The winter wheat fields without irrigation facilities and relying on natural precipitation were defined as the rainfed farmland, and the number of samples was 28. The irrigated fields equipped with irrigation facilities and are irrigated according to the growth regularity of winter wheat and natural precipitations were defined as irrigation field, and the number of samples was 31. To obtain a wide range and diverse LAI, all samples were collected at the joining stage, booting stage and filling stage of winter wheat. Thus, 93 and 84 samples for irrigated and rainfed field, respectively, were collected in this study.

### Canopy spectra measurement

The canopy reflectance of winter wheat was obtained with an ASD spectroradiometer (Analytical Spectral Devices, Inc. (ASD), USA) under cloudless conditions and as close to solar noon as possible. The wavelength range was 350 ~ 2500 nm at a field angle of 25°, the spectra sampling interval is 1.4 nm between 350 ~ 1000 nm with the spectral resolution of 3 nm; the spectral sampling interval is 2 nm between1000 ~ 2500 nm with the spectral resolution of 10 nm. The canopy spectral measurement was performed during sunny, windless or low wind speed condition with a measuring time of 10:00 ~ 14:00. When measuring sensor probe is vertically down, the vertical height to the top of the canopy is about 1.5 m, and the diameter of ground field view is 0.44 m. Measurements were repeated three times for each sample, and the average was taken as the spectral reflectance at that point. Standard whiteboard correction was adopted in the measurement process. (Standard whiteboard reflectivity is 1, the target object spectrum measured is the dimensionless relative reflectivity)

### Measurement of leaf area index

In study, we adopted the reported method to measure the LAI [17]. 1 m^**2**^ of winter wheat was collected at the spectrum measurement area. After the stem leaves were collected, five leaves were randomly selected from all the leaves, and were arranged in a row (the middle veins of the leaves were close together). The total width of the middle vein of the five leaves was accurately measured with a ruler, and 4 cm of the middle vein was cut accurately. The area of the leaf was obtained. Then we measured leaf area of all leaves for 1 m^**2**^ (S′). Then the total area of the leaves can be obtained by using the following equation.

$$S=S_{1}\times \frac{W_{1}+W_{2}}{W_{1}}$$

$$LAI=\frac{S}{S'}$$

Where, S and S_1_ are the area of all leaves for 1 m^2^ and five cut leaves, respectively. W_1_ and W_2_ are the weight of all leaves for 1 m^2^ and five cut leaves, respectively.

### Spectral analysis

In this paper, we evaluated the spectral bands of LAI by using SPA method and constructed a LAI prediction model based on evaluated bands with MLR method. As the special function of PLSR on overcoming the co-linearity and realizing the dimension reduction for hyperspectral data, it was widely applied in many fields and it is especially used when the number of independent variables is far more than the sample number. Therefore, we adopted the SPA-MLR and PLSR to monitor the LAI model and the models performance was compared and analyzed to study the effects of rainfed fields and irrigated fields on predicting LAI of winter wheat.

By analyzing the correlation between LAI and canopy spectra, we can indirectly confirm and select the important wavebands. In addition, the two parameters of VIP and B-coefficient parameter derived from the PLSR method can statistically characterize the impact of the independent variable on dependent variable. Generally, it always indicates that the independent variables have a greater contribution and influence on dependent variable when the VIP > 0.8, and the absolute value of B-coefficient is greater as well. Then, the wavelengths with high VIP and absolute value of B-coefficient can be considered as sensitive with LAI. Therefore, the sensitive wavelengths were multi-anglely determined with correlation analysis, PLSR, SPA, and the multi-variate statistical methods of PLSR and SPA-MLR were applied on constructing the predictive models of LAI in study.

### Model evaluation

The performance of the model was evaluated by using coefficient of determination (R^2^), root mean squared error (RMSE) and Residual Test Deviation (RPD). Among them, R^2^ and RMSE represent the prediction accuracy of the model; the RPD reflects the stability of the model. It is generally believed that when RPD is > 2, the prediction ability of the model is better; when it is between 1.4 and 2, it indicates that the model has a moderate predictive ability; when RPD <1.4, it indicates that the model predictive ability is poor [18].

### Data processing

Raw spectral data was smoothed by using Savitzky–Golay method with 8 points, and the spectral region between 400–1400 nm was selected for further analysis in study as the selected spectral region was high related to crop growth. The origin 8.0 software (OriginLab, USA) was used to perform 8-point smoothing and mapping, and the correlation analysis. SPA and PLSR were performed with Matlab 7.0 (MathWorks, USA).

## Results

### Descriptive statistical analysis of LAI

In this study, all the samples were randomly divided to calibration set and validation set. There were 62 and 31 sample data in calibration set and validation set for irrigated fields; 56 and 28 sample data were used as the calibration set and validation set for rainfed fields. The results of LAI descriptive statistical analysis of all data sets are shown in Table 1.

**Table 1:** Descriptive statistic analysis of leaf area index.

Field style	Sample classifications	Sample	Range	Minimum	Maximum	Average	Standard deviation
Irrigation fields	Calibrated set	62	10.60	1.31	11.91	5.100	2.283
	Validation set	31	7.31	1.53	8.84	5.248	2.094
Rainfed fields	Calibrated set	56	8.95	0.31	9.27	2.951	2.002
	Validation set	28	4.64	0.28	4.92	2.458	1.428

According to the minimum value, the maximum value and the average value of the leaf area index (LAI) of the winter wheat in the rainfed and irrigated fields in Table 1, the growth of winter wheat in the irrigated field was better than that in the rainfed winter wheat. Depending on the parameters of standard deviation (SD) and range, the growth of winter wheat in irrigated fields and had a significant variance. In addition, the calibration set and validation set data of winter wheat leaf area index in rainfed fields and irrigated fields accord with the statistical requirements, and can be further evaluated based on the statistical analysis.

### Correlation analysis

**Fig 1:**
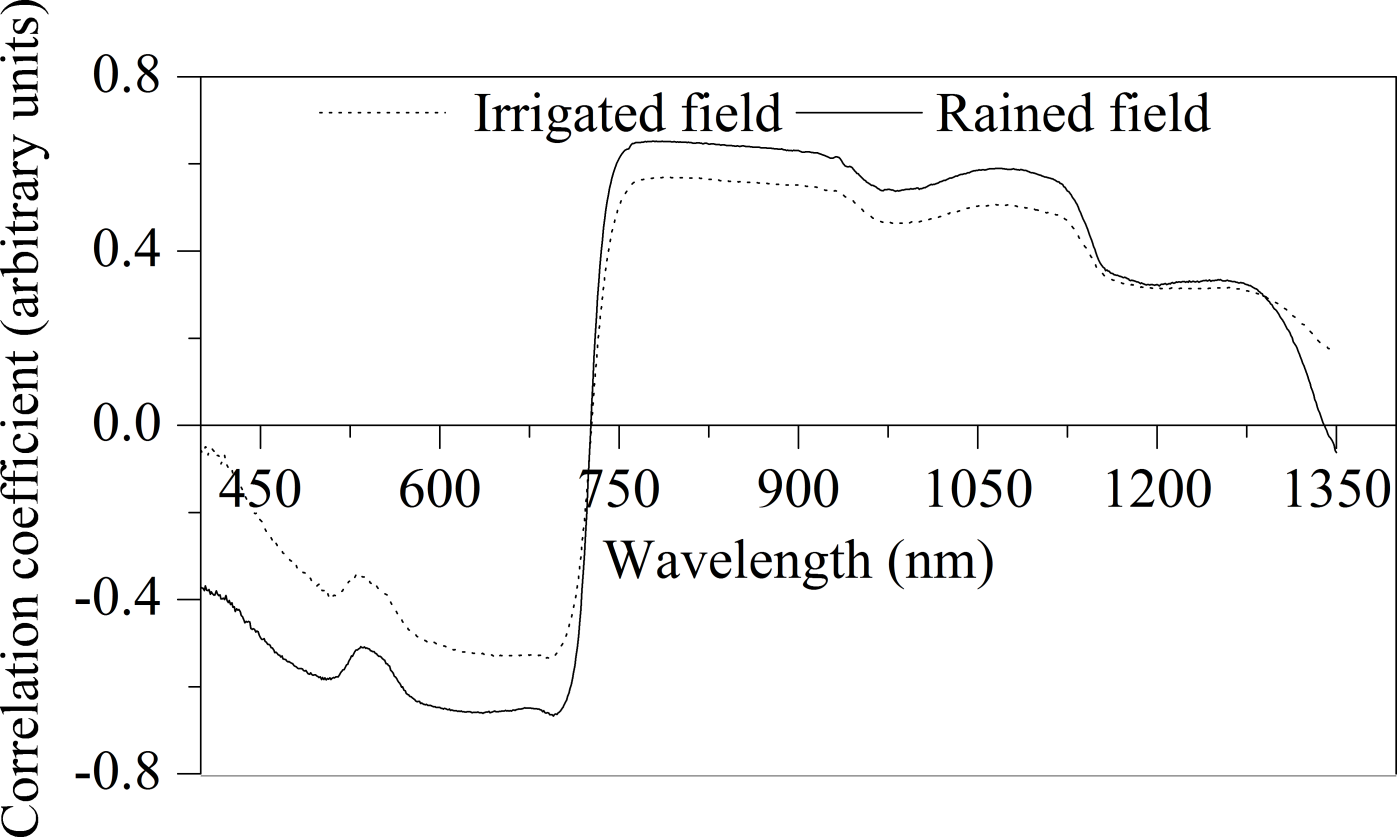
Correlation coefficients between spectral wavebands and LAI of winter wheat in rainfed field and irrigated field.

Correlation analysis was performed between the spectral region from 400 to 1400 nm and LAI, and the correlation coefficient curve was drawn as shown in Fig 1. It has been shown that the correlation between the LAI and canopy spectra of winter wheat in both the fields is good, as the correlation coefficient reached to 0.65. These results indicate that there is a close relationship between the LAI and spectral wavelengths and it is feasible to monitor the LAI of winter wheat by using the spectroscopy technology. The correlation of LAI and spectrum of winter wheat in both the fields are similar, however, there are little differences, which are mainly caused by the difference of winter wheat growth in rainfed fields and irrigated fields. In addition, the Fig 1 also shown that the correlation coefficient of LAI and spectrum of winter wheat in rainfed fields was higher compared to that in irrigation fields.

### Extraction of sensitive wavelengths for leaf area index

#### SPA method

The spectral wavebands of LAI evaluated by successive projections algorithm (SPA) were listed in Table 2. 10 bands were selected in rainfed and irrigated fields, most of which were concentrated in the visible and red edge area. Only 1120, 1290, 816 and 1127 nm are in the near infrared band. In rainfed fields, the selected spectral bands are evenly distributed in both the visible and near-infrared regions, whereas for irrigated fields, 50% of them were centered at 400–450 nm in the visible region, because the growth of winter wheat was better in irrigated fields, and moreover, the spectrum absorption of chlorophyll was stronger. Physiologically, the correlation between chlorophyll and LAI was positive. On the other hand, the selected bands are similar, such as 404, 406, 679, 727 and 1120 nm of the LAI of rainfed farm land and 404, 407, 677, 735 and 1127 nm of the LAI of the irrigated field, among which 679,727 nm in the rainfed field and 677,735 nm in the irrigated field are located in the red edge area.

**Table 2:** Important wavelengths of LAI selected with the SPA method using the calibration set.

Field style	Important wavelengths (nm)
Irrigation field	404、407、413、417、450、677、715、735、816、1127
Rainfed fields	404、406、432、501、540、679、727、779、1120、1290

#### PLSR method

**Fig 2:**
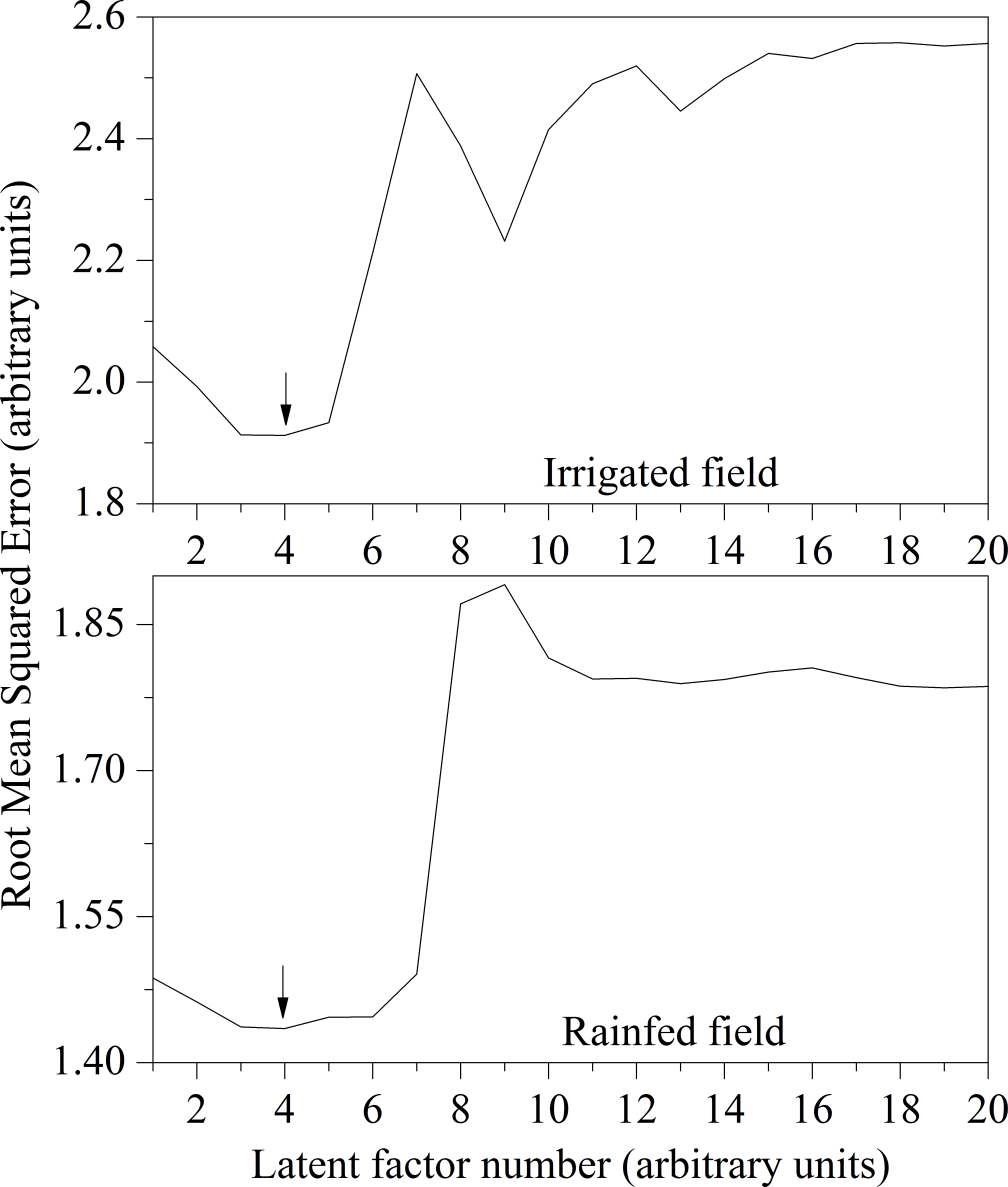
Selection of the optimal factor number of LAI in PLSR analysis under the rainfed field and irrigated field.

The method of cross validation (leave-one-out) was applied to validate the model performance under different factor number, and the smallest value of RMSE was used to select the optimal factor number and its corresponding PLSR model (Fig 2).

**Fig 3:**
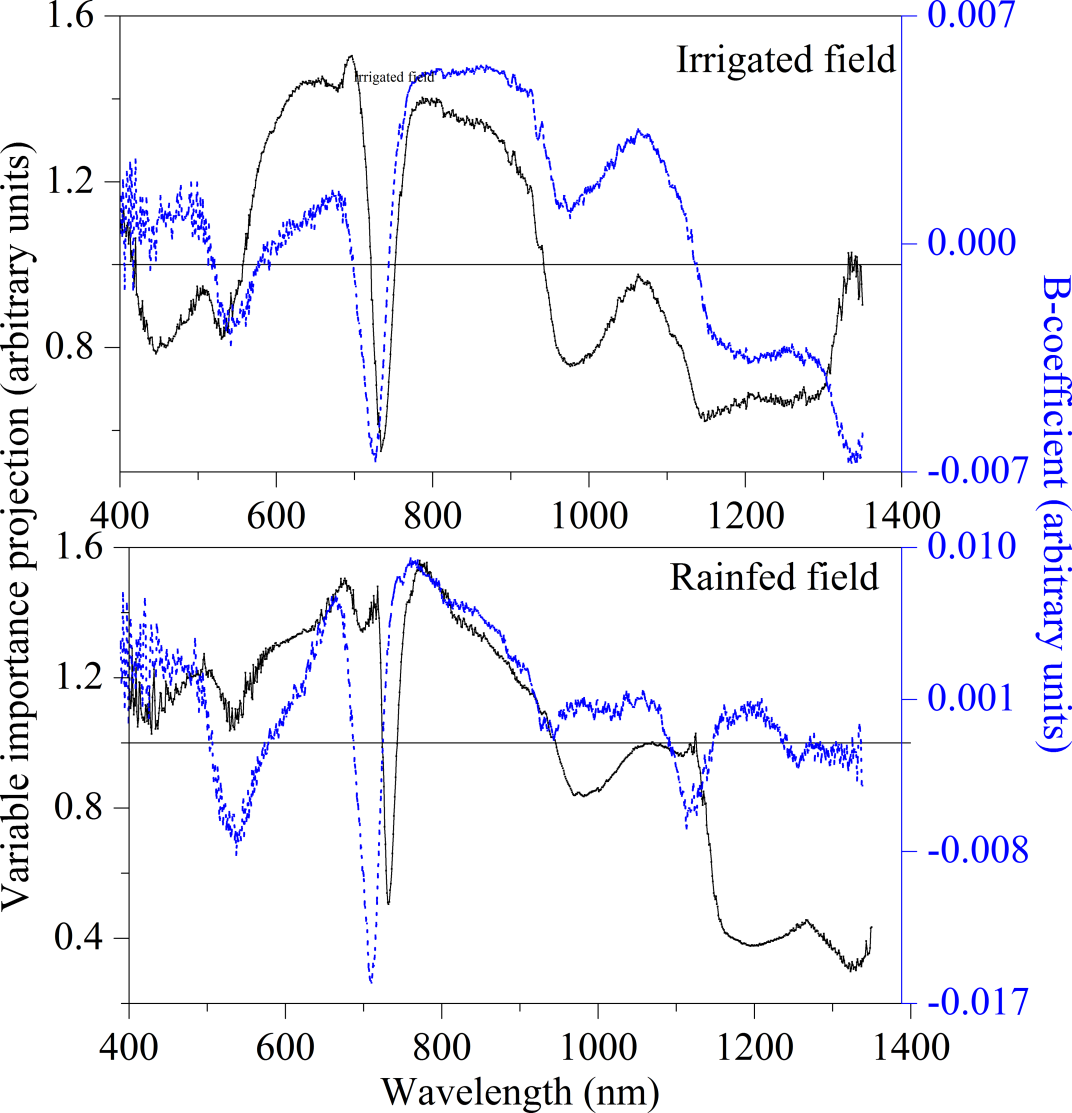
Variable characteristic of LAI for winter wheat derived from PLSR analysis in rainfed field and irrigated field.

From the results, it has been observed that the RMSE of the model for both the rainfed and irrigated fields is the small when the factor number is 4. And then, the B-coefficient and VIP for the selected number of factors are evaluated accordingly, and the results are shown in Fig 3.

According to contribution of independent variables to the PLSR model by using the B-coefficient and VIP parameters derived from PLSR analysis, we could see that under the conditions of irrigated field, except for the spectral regions like 710–750 nm, 960–1010 nm and 1110–1310 nm, the VIP values of all other regions were greater than 0.8. In the rainfed field, the VIP values of the spectral regions 720–750 nm and 1130–1350 nm were less than 0.8, indicating that the contribution of this region to the model was less, because the B-coefficient value between 710 and 750 nm is the smallest, this region still has certain influence on the model. The result of PLSR analysis would supply some references in determining the important wavelengths of LAI in study.

### Monitoring models of LAI

The prediction model of LAI was constructed by combining the spectral bands and MLR methods extracted with SPA method and compared with the full-band model of PLSR method. The performance of calibrated and validated models for both the rainfed and irrigated fields constructed with multivariate method were shown in Table 3.

**Table 3:** Models performance of LAI based on the multivariate methods in rainfed field and irrigated field.

Multivariate methods	Field styles	Calibration set			Validation set			Variable number
		R^2^	RMSE	RPD	R^2^	RMSE	RPD	
SPA-MLR	Irrigation field	0.716	1.059	1.538	0.635	1.221	1.469	10
	Rainfed field	0.736	1.169	1.624	0.661	1.206	1.473	10
PLSR	Irrigation field	0.552	1.520	1.413	0.541	1.742	1.084	4
	Rained field	0.579	1.288	1.456	0.684	1.122	1.400	4

Table 3 shows that the LAI prediction models (SPA-MLR) for both of the fields was performed with higher estimation accuracy (R^2^>0.716, RMSE<1.169, RPD>1.538; R^2^>0.635, RMSE<1.221, RPD>1.469, for calibrated models and validated models, respectively). However, the PLSR model of LAI based on the full spectrum had moderate prediction (R^2^<0.579, RMSE>1.288, RPD<1.456). The dare also showed that the stability of the prediction model wasn’t robust for the rainfed field with RPD <1.4. Comparing the model performance for the methods of SPA-MLR and PLSR, we conclude that the SPA-MLR had higher predictive accuracy.

**Fig 4:**
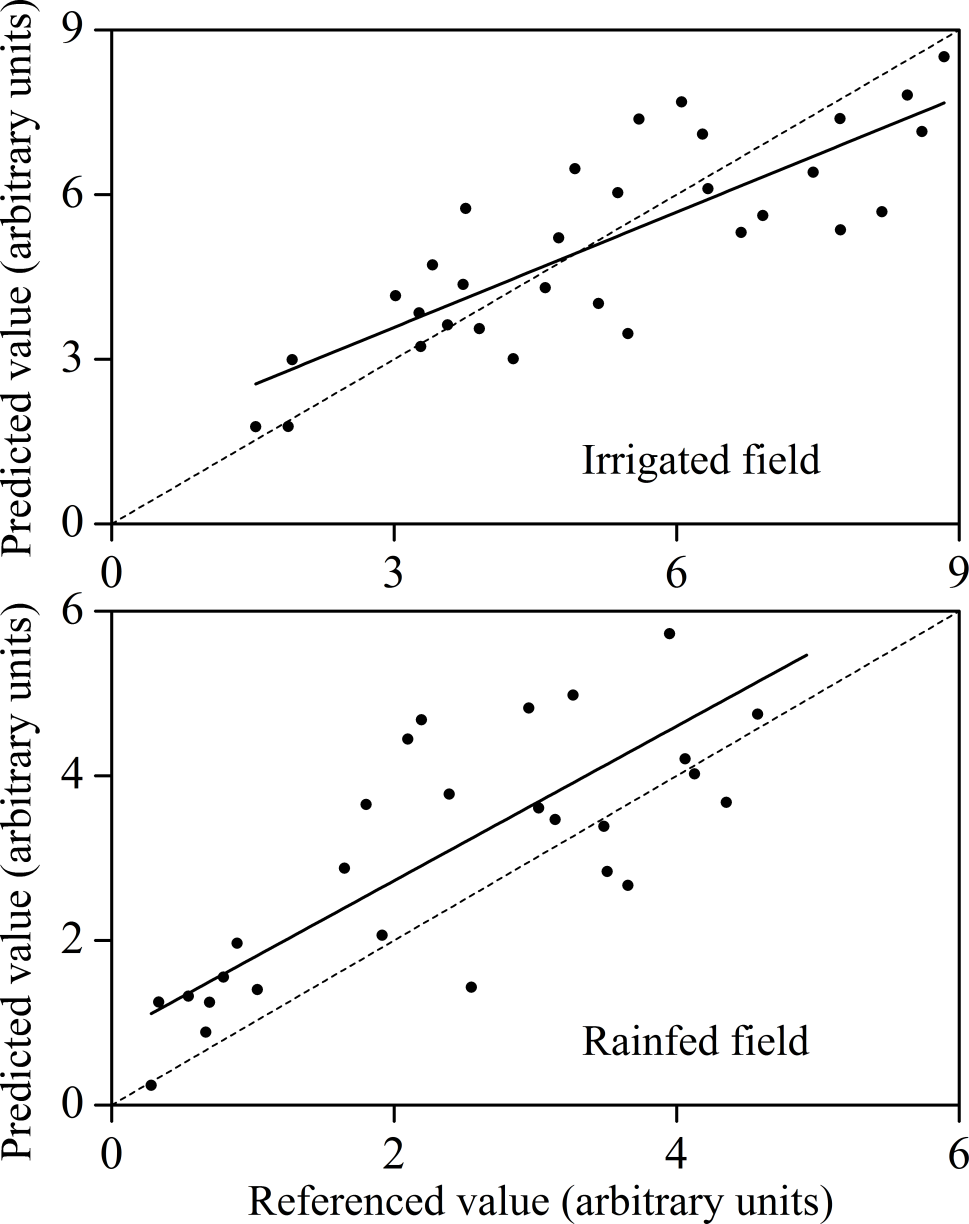
Linear relationships (1:1) between the referenced values and predicted values of LAI in irrigated field and rained field of winter wheat (the solid line and the dotted line are the fitted line and referenced line, respectively).

Based on the SPA-MLR method, the relationship between the predicted value and the measured value for the rainfed and irrigated fields was plotted (Fig 4). Although the PLSR model has higher R^2^ and lower RMSE in the irrigated fields, the practical application of this model still has some limitations due to the low prediction accuracy of the calibration model. In terms of the impact of rainfed and irrigated fields on LAI estimation, the rainfed fields are superior to that of the irrigated field.

## Discussion

### Spectral characteristics

In this study, we analyzed the correlation between LAI and canopy spectral reflectance of winter wheat in rainfed fields and irrigated fields, and found that there was a high correlation between spectral band and LAI and where the correlation coefficient of some bands was even higher than 0.65. The results laid the foundation to further explore the spectral information of the LAI and to construct the spectral monitoring model with high accuracy. However, the correlations between LAI and spectrum of winter wheat in rainfed fields and irrigated lands were different. The correlation between LAI and canopy spectrum of rainfed farmland is higher than that of irrigated field. Since the spectral bands that make up the vegetation index are saturated under high vegetation conditions (higher LAI), the sensitivity to LAI is reduced. From the point of view of the mechanism of spectroscopy to monitor the crop, under low vegetation coverage (lower LAI), the visible light and near infrared bands are sensitive to the growth of the crop. When the vegetation coverage or growth reaches a certain level, the sensitivity to LAI will be reduced. In the present study, the average level of LAI in irrigation field was greater than 5; while it is lower than 3 for those in rainfed farmland (Table 1). Wang et al. [19] reported that when LAI>3, the canopy spectrum of winter wheat would undergo a "saturation" phenomenon, which would reduce the response sensitivity of the spectrum on LAI. In present study, although the growth of winter wheat in irrigated field is better than that in rainfed field, the correlation between LAI and the spectrum in irrigated field was affected by the "saturation" effect, and finally this phenomenon produced a negative effect on the model performance.

### Selection of important wavelengths

In contrast selected spectral wavelengths of LAI in rainfed and irrigated land by using the SPA method, we found that except for 2 bands (1120, 1290 nm of rainfed field and 816,1127 nm of irrigated field) distributed in the near-infrared region, the remaining spectral bands were concentrated in the visible and red edge regions. Spectral bands (404, 406, 679, 727 and 1120 nm) of rainfed field were similar to those of irrigated land (404, 407, 677, 735 and 1127 nm). It indicates that these bands are closely related to LAI. Delegido et al. [20] suggested that there is a close relationship between the chlorophyll and spectral regions of 400–500 nm and 600–760 nm. Feng et al. [5] demonstrated that the LAI had an important correlation with the spectral regions of 350–510 nm and 571–716 nm. Zhang et al. [21] reported that 1120 nm was sensitive to leaf moisture. The selected spectral bands of rainfed field are evenly distributed in the visible and near-infrared region, however, for irrigated field, 50% of the selected bands were concentrated in 400–450 nm in the visible region, and this could be due to the fact that the growth of the winter wheat is better under the irrigated conditions, and the chlorophyll content of leaves is higher, which made it easier to absorb light [22]. Physiologically, leaf pigment has a close relationship with LAI, which leads to a good relationship between the above said spectral bands and LAI. In addition, 679 and 727 nm in rainfed fields and 677 and 735 nm in irrigated fields are all located in the red-edge area, and many studies have shown that red-edge is closely related to LAI, which is widely used in winter wheat growth monitoring [23].

Comparing the selected wavelengths evaluated by SPA method and the correlation analysis result, it showed that the selected spectral bands 501, 540, 1120 and 1290 nm in the rainfed field and 1127 nm in the irrigated field had an extreme correlation coefficient. 679, 729 and 719 nm in the rainfed field and 677, 715 and 816 nm of the irrigated land were located at the maximum of the correlation coefficient, and the 735 nm of the irrigated land appeared at the mutation point of the correlation coefficient. Previous studies have confirmed that these spectral characteristic bands are generally located at the extreme or mutation point of the correlation coefficient [24–26]. The selective bands (404, 407, 413, 417 and 450 nm) of irrigated land were not consistent with the above results. However, the fact that the chlorophyll had a strong absorption or reflectance at these points under irrigation condition [8], and the positive correlation between chlorophyll and LAI might lead to the current situation.

In addition, according to the B-coefficient and VIP as evaluated by PLSR analysis, the value of VIP and B-coefficients at the bands of 404, 407, 413, 450, 677, 735 and 816 nm in the spectral bands of the irrigated fields were high. The VIP value is lower than 0.8 at the 735 nm, however, its B-coefficient is close to -0.007, which indicates that 735 nm had a great effect on the PLSR model. For rainfed fields, the VIP and B-coefficient were also high at the wavelengths of 404, 406, 432, 501, 540, 679 and 1120 nm, where the VIP is greater than 0.8. The maximum value was observed at the bands of 779 nm where VIP and B-coefficient reached to 1.5506 and 0.0092, respectively. The wavelengths of 735 nm in irrigated land and 727 nm in rainfed condition were the common wavelengths belonging to the red edge area, which indicates that the red edge point was closely related with the LAI.

### LAI monitoring model

Many researchers have comparatively analyzed the different modeling methods of winter wheat growth variables [27–29]. In this study, we made an attempt to evaluate the important spectral bands of LAI in rainfed and irrigated conditions by using the SPA method, and to construct the estimation model of LAI based on spectral characteristic bands combining the MLR method. In contrast, we further constructed the PLSR model of LAI based on the full spectrum to comprehensively evaluate the application of the multivariate statistical analysis on monitoring the LAI of winter wheat.

In our study, the SPA-MLR model of LAI achieved better performance (R^2^ = 0.736, 0.716; RMSE = 1.169, 1.059; RPD = 1.6245, 1.538, for rainfed farmland and irrigated land, respectively) based on the important wavelengths. According to the model classification by Chang et al. [18], these models obtained a moderate predictive performance (RPD> 1.4). The validation result showed that the validated model reached a moderate prediction level (rainfed field: R^2^ = 0.661, RMSE = 1.206, RPD = 1.473; irrigated land: R^2^ = 0.635, RMSE = 1.221, RPD = 1.469), which indicates the accuracy of the spectral band evaluated by the SPA method and the stability of the model constructed by the SPA-MLR method. Compared with the performance of PLSR models which consisted of the full spectrum, showed that the accuracy of the models was lower than that of SPA-MLR method. In previous studies, the PLSR method is superior to the MLR method in modeling accuracy and model robustness [30], however, contrast results were obtained in this study. The PLSR model consists of four potential factors, while the SPA-MLR model had 10 important spectral bands. The more the independent variables in the model, the higher the prediction accuracy would be. In the SPA-MLR models, the independent variables are composed of important spectral bands selected by the SPA method. These selected bands could represent the information of LAI and had a close relationship with LAI [11]. However, the PLSR model was based on full-spectrum information and many of the spectral information were irrelevant to the LAI. Thus the irrelevant would definitely affect the accuracy of the model. Moreover, 10 spectral bands were input into the SPA-MLR models and the model was easier to be interpreted and applied in practice [12, 31, 32]. In case of PLSR model, although composed of four factors, the complexity of the model was still high as each factor was related with the whole wavelengths.

Comparing the LAI monitoring models in rainfed field and irrigated land, we could conclude that the LAI model of winter wheat in rainfed farmland is superior to that in irrigated land. In this study, the canopy spectrum of winter wheat was more easily affected by "saturation" phenomenon, which definitely decreased the sensitivity of the spectrum and LAI, and ultimately affected the accuracy and stability of the predictive model of LAI [19]. Finally, it leaded to the fact that the LAI model accuracy of winter wheat under the irrigated field is lower than that of rainfed field. At the same time, the overall correlation between the LAI of winter wheat in rainfed field and its canopy spectral is higher than that of irrigated field, which also indirectly explains the difference of model performance.

The prediction accuracy of the LAI model constructed in this study was lower than previous studies (R^2^ <0.74, RPD <1.65), which is mainly due to the fact that the spectral data are easily influenced by the soil background, environmental factors and winter wheat varieties, especially in field farmland. These factors inevitably increased the heterogeneity of the sample data and produced a negative effect on model performance. However, considering the models of LAI developed under different irrigated situations were constructed and the validation models obtained medium prediction accuracy, the LAI estimation model constructed in our study still had certain applicability in practice. Moreover, we reduced the spectral range from nearly 1000 to 10 using SPA algorithm to realize the data reduction, and then we combined the MLR method to construct the LAI linear model based on the selected spectral bands determined with the PLSR analysis and correlation analysis. These procedures including the determination of important wavelengths and construction of monitoring model, and the application of multivariate statistical method in spectroscopy field would provides a theoretical reference and practical exploration for spectral variables selection and model construction.

Although this study made an good achievement in monitoring the LAI of winter wheat in rainfed and irrigated land, the classification of rainfed and irrigated land needs further subdivision, the sampling period needs to be more complete, the range and quantity of sampling points should be expanded, and the variable selection and model construction method should be further explored in order to realize the spectrum monitoring of winter wheat growth in rainfed and irrigated fields.

## Conclusion

In this study, we considered the winter wheat in rainfed and irrigated fields in Wenxi county as the research object, to analyze the differences of spectral response under rainfed and irrigation farmland conditions. The results indicated that monitoring the LAI research of winter wheat in rainfed and irrigated farmland was necessary and significant by using the spectroscopy technology. The dimension reduction of spectral data was analyzed by using SPA method and the important spectral bands of LAI of winter wheat in rainfed field and irrigated field were determined as 404, 407, 413, 417, 450, 677, 715, 735, 816, 1127 nm and 404, 406, 432, 501, 540, 679, 727, 779, 1120, 1290 nm for irrigated field and rainfed fields, respectively. The predictive models of LAI constructed with SPA-MLR were obtained (rainfed field: R^2^ = 0.736, RMSE = 1.169, RPD = 1.6245; irrigated field: R^2^ = 0.716, RMSE = 1.059, RPD = 1.538). These procedures including the determination of important wavelengths and construction of monitoring model, and the application of multivariate statistical method in spectroscopy field would provide a theoretical reference and practical exploration for spectral variables selection and model construction.
